# Design, Synthesis, Biological Evaluation, and Computational Studies of Novel Tri-Aryl Imidazole-Benzene Sulfonamide Hybrids as Promising Selective Carbonic Anhydrase IX and XII Inhibitors

**DOI:** 10.3390/molecules26164718

**Published:** 2021-08-04

**Authors:** Lamya H. Al-Wahaibi, Bahaa G. M. Youssif, Ehab S. Taher, Ahmed H. Abdelazeem, Antar A. Abdelhamid, Adel A. Marzouk

**Affiliations:** 1 Department of Chemistry, College of Sciences, Princess Nourah Bint Abdulrahman University, P.O. Box 84428, Riyadh 11564, Saudi Arabia; 2 Pharmaceutical Organic Chemistry Department, Faculty of Pharmacy, Assiut University, Assiut 71526, Egypt; 3 Department of Pharmaceutical Organic Chemistry, Faculty of Pharmacy, Al-Azhar University, Assiut 71524, Egypt; ehaborganic@yahoo.com; 4 Department of Medicinal Chemistry, Faculty of Pharmacy, Beni-Suef University, Beni-Suef 62514, Egypt; ahmed.abdelazeem@pharm.bsu.edu.eg; 5 Department of Pharmaceutical Sciences, College of Pharmacy, Riyadh Elm University, Riyadh 11681, Saudi Arabia; 6 Department of Chemistry, Faculty of Science, Sohag University, Sohag 82524, Egypt; drantar25@yahoo.com; 7 Chemistry Department, Faculty of Science, Albaha University, P.O. Box 1988, Albaha 65731, Saudi Arabia; 8 Department of Pharmaceutical Chemistry, Faculty of Pharmacy, Al-Azhar University, Assiut 71524, Egypt; adelmarzouk@azhar.edu.eg

**Keywords:** imidazole, benzene sulfonamide, carbonic anhydrase IX, XII

## Abstract

A novel series of tri-aryl imidazole derivatives **5a**–**n** carrying benzene sulfonamide moiety has been designed for their selective inhibitory against hCA **IX** and **XII** activity. Six compounds were found to be potent and selective CA IX inhibitors with the order of **5g** > **5b** > **5d** > **5e** > **5g** > **5n** (Ki = 0.3–1.3 μM, and selectivity ratio for hCA IX over hCA XII = 5–12) relative to acetazolamide (Ki = 0.03 μM, and selectivity ratio for hCA IX over hCA XII = 0.20). The previous sixth inhibitors have been further investigated for their anti-proliferative activity against four different cancer cell lines using MTT assay. Compounds **5g** and **5b** demonstrated higher antiproliferative activity than other tested compounds (with GI_50_ = 2.3 and 2.8 M, respectively) in comparison to doxorubicin (GI_50_ = 1.1 M). Docking studies of these two compounds adopted orientation and binding interactions with a higher liability to enter the active side pocket CA-IX selectively similar to that of ligand **9FK**. Molecular modelling simulation showed good agreement with the acquired biological evaluation.

## 1. Introduction

**Figure 1:**
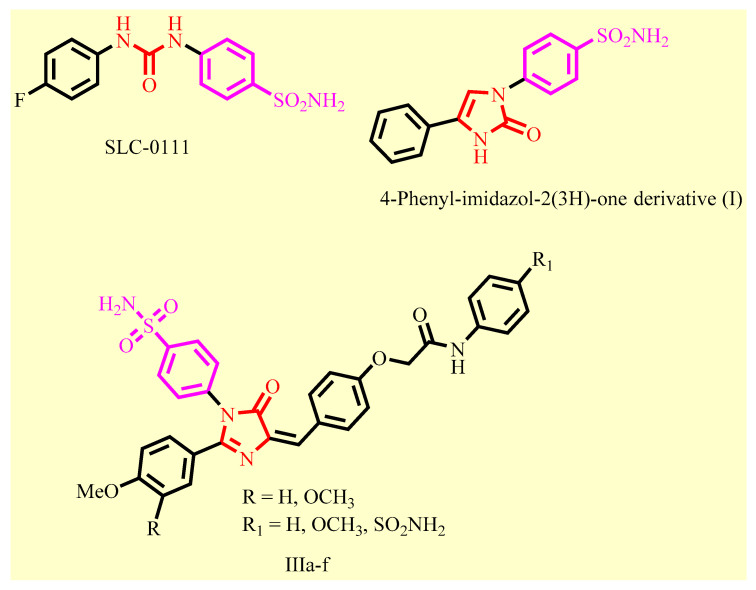
Structure of **SLC-0111**, compound **I** and **IIIa–f**.

Carbonic anhydrases (CAs) are metalloenzymes that catalyze the reversible hydration of CO_2_ into protons and bicarbonate [1]. CAs are involved in many physiological and pathological processes, including respiration and the transport of CO_2_ and bicarbonate between metabolizing tissues and the lungs, electrolyte secretion in various tissues and organs, biosynthetic reactions bone resorption, calcification, and tumor growth. [1]. As a result, several CA isoforms involved in these processes are important therapeutic targets, and their inhibitors have a high potential for pharmacological intervention in a variety of diseases such as glaucoma, obesity, osteoporosis, and cancer [2,3]. There are now 16 different mammalian—CA isoforms recognized, including human isoforms [4]. These CAs are classified as cytosolic (CA I, CA II, CA III, CA VII, and CA XIII), membrane bound (CA IV, CA IX, CA XII, CA XIV, and CA XV), and mitochondrial (CA VA and CA VB). Cytosolic hCA I and II are the most common isoforms, and inhibitors of these isoforms are primarily utilized as antiglaucoma, diuretics, antiepileptic, antiedema medications, and for the treatment of altitude sickness [1,4,5]. However, in recent years, cancer research has mostly focused on the hCA IX and XII isoforms [6,7]. CA IX is a transmembrane isoform found in the GI mucosa and malignancies [8]. CA IX is involved in a number of functions, including carcinogenesis, pH control, tumor cell proliferation and migration, and cell adhesion. CA IX expression is regulated by tumor hypoxia, which has been identified as a risk factor for the progression of a variety of malignancies [9,10]. CA IX inhibition could be a promising anticancer therapeutic target. Similarly, the CA XII isoform has been linked to the progression of many cancers [11]. CA XII is a transmembrane isoform found in a variety of organs including the eye, malignancies, prostate, ovaries, intestines, and kidney. Because of its expression in numerous organs, it has been linked to a variety of malignancies [12,13]. Because hCA IX/XII have been shown to play a significant role in tumor growth and metastasis, they have been identified as promising targets for the development of novel antineoplastic drugs. The biggest impediment to developing new cancer therapies by targeting hCA IX/XII occurred as a result of nonselective inhibition of hCA I/ II isoforms, which was thought to be the main cause of the undesired side effects reported with these nonselective inhibitors [1,5]. The majority of the investigated inhibitors against one or more hCAs are sulfonamide-containing aromatic/heterocyclic compounds, where the sulfonamide moiety acts as a zinc binding group (ZBG) and the heterocyclic scaffold is thought to selectively promote interactions with isoform unique residues at the active site cavity [5,14]. SLC-0111 (Figure 1), a sulfonamide derivative with strong selectivity against the CA IX isoform, has entered clinical trials as a single agent for solid tumors [15] or as part of a combination therapy for pancreatic ductal carcinoma [16].

In recent years, the aryl imidazole scaffold has gained recognition as a helpful and promising scaffold for the design and development of effective CA inhibitors. The synthesis and biological evaluation of novel 4-phenyl-imidazol-2(3*H*)-one derivatives as CA inhibitors were described by Congiu et al. [17], (Compound **I**, Figure 1). These imidazole compounds were discovered to be selective nanomolar inhibitors of CAs IX and XII. Another class of 1,2,4-trisubstituted imidazoles with a 4-benzylidene moiety and primary and secondary sulfonamide groups that act as dual carbonic anhydrase (isoforms I, II, IV, and IX) and p38-MAPK inhibitors were designed and synthesized (**IIa–f** in Figure 1) [18]. The results of this study’s SAR revealed that the presence of a primary sulfamoyl group is vital for the CA inhibitory activity.

**Figure 2:**
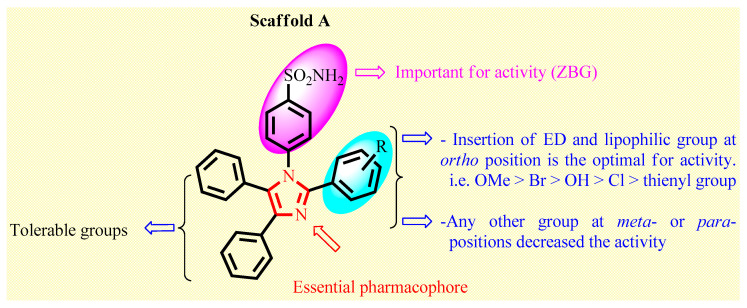
Rational design and structural modification of **Scaffold A**.

Encouraged by the facts mentioned above and mindful of the significance of continuous development of selective hCA IX inhibitors to feed into anticancer discovery pipeline, we report the synthesis of a new series of 2,4,5-triphenylimidazole-benzensulfonamides **5a**–**n** (**Scaffold A**, Figure 2) using a one-pot single-step multicomponent reaction including primary aromatic amine (sulfanilamide), aldehydes, and benzil. The newly synthesized compounds contain primary sulfonamide group as Zinc-binding group (ZBG). The substitution pattern on the pendant 2-aryl moiety was chosen to provide a variety of lipophilic and electronic environments that would affect the CA inhibitory action of **5a**–**n**. All the synthesized imidazoles **5a**–**n** were characterized and biologically tested against the physiologically relevant hCA isoforms, hCA I, II (cytosolic), as well as hCA IX and XII (transmembrane, tumor-associated isoforms) using stopped-flow CO_2_ hydrase assay. A molecular docking study for the prepared imidazoles within CA IX active sites was carried out to rationalize the obtained results. Moreover, the most potent and selective hCA IX inhibitors **5b**, **5d**, **5e**, **5g**, **5j** and **5n** were assessed for their antiproliferative activity against four different cancer cell lines.

## 2. Results and Discussion

### 2.1. Chemistry

**Scheme 1:**
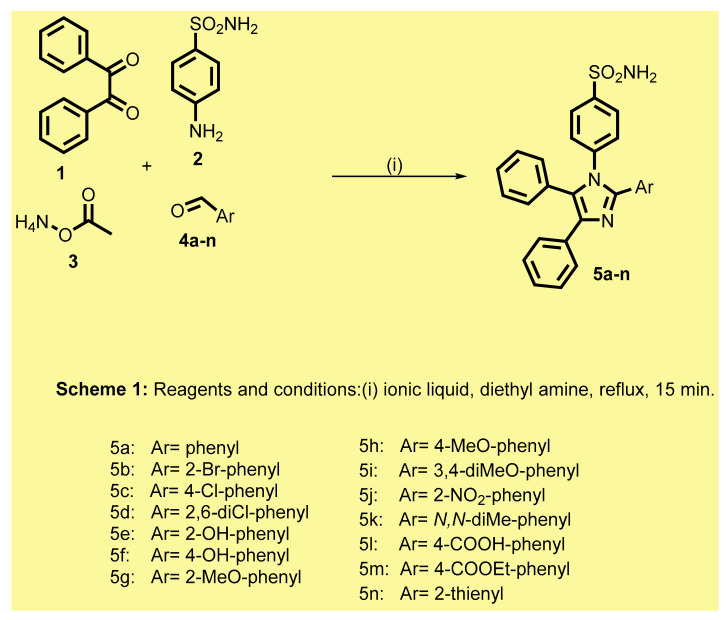
Synthesis of compounds **5a**–**n**.

Tri-aryl imidazole-benzene sulphonamide hybrids **5a**–**n** were synthesized by adopting a one-pot single-step multicomponent reaction involving primary aromatic amine (sulfanilamide), aldehydes **4a**–**n**, benzil, and ammonium acetate in the presence of diethyl ammonium hydrogen sulfate (ionic liquid) as shown in Scheme 1.

With simple work-up processes and good yields of the target compounds, the reaction time ranged from 15 to 30 min. All the newly synthesized compounds were analyzed by IR, ^1^H NMR, ^13^C NMR, and elemental analyses. In the ^1^H NMR spectra of chemical **5i** (Ar = 3,4-dimethoxyphenyl), two singlet signals were common for methoxy groups: one at 3.52 ppm (OCH_3_) and the second at 3.76 ppm (OCH_3_) protons, as well as a doublet signal at 7.77–7.79 of 2H associated to NH_2_ of the sulfonamide moiety. Element analyses were used to confirm the purity of the produced compounds, and the results agreed with the products’ molecular formula.

### 2.2. Biology

Newly synthesized hybrids **5a**–**n** were tested for their inhibitory activity against four physiologically and pharmacologically CA isoforms, namely cytosolic hCA I and II, and tumor-associated isoforms hCA IX and XII, using a CO_2_ hydrase stop-flow assay [19]. Acetazolamide (AAZ), clinically used sulphonamide, was used as the standard drug. The inhibition data of **5a**–**n** were cited in Table 1. Generally, **5a**–**n** compounds have not been shown to inhibit the cytosolic isoforms hCA I and hCA II (Ki > 100 μM). Compounds **5a**–**n** showed a number of inhibitory profiles against the transmembrane tumor-associated isoform hCA IX. Compounds **5g**, **5b**, **5d**, **5e**, **5j** and **5n** had sub micromolar inhibitory potencies against hCA IX (Kis) of 0.32, 0.36, 0.72, 0.84, 1.10, and 1.3 μM, respectively, with a pronounced selectivity for hCA IX over hCA XII (12, 11.50, 7.00, 6.50, 5.60, and 5.0, respectively) in comparison to AAZ (selectivity ratio for hCA IX over hCA XII = 0.20) [20,21]. In addition, compounds **5a**–**n** displayed a variable degree of inhibition against the other transmembrane tumor-associated isoform, hCA XII, with Kis below 7 μM for all these compounds. Among these compounds, compounds **5g** (Ar = 2-MeO-phenyl) and **5b** (Ar = 2-Br-phenyl) showed promising inhibitory activity with a Ki value of 3.7 and 4.6 μM, respectively. The *ortho* substitution in the aryl moiety appears to correlate with higher inhibitory effects against both hCA IX and XII. Notably, the activity increased with (Ar) in the order of 2-MeO-phenyl > 2-Br-phenyl > 2-HO-phenyl > 2-NO_2_-phenyl > 2-thienyl. In comparison to **5e** (Ar = 2-HO-phenyl, Ki = 0.8 μM), compound **5f** (Ar = 4-HO-phenyl) had a 10-fold lower inhibitory activity on hCA IX (Ki = 8.4 M). The similar pattern holds true for **5g** (Ar = 2-MeO-phenyl) and **5h** (Ar = 4-MeO-phenyl), with **5h** being 22-fold less reactive than the *ortho*-derivative. From these results, **5a**–**n** derivatives exhibited selective inhibition of hCA IX and XII compared to hCA I and II in the micromolar range.

Finally, the inhibitory activities of the newly synthesized **5a**–**n** against hCAs could serve as a preliminary study for future biological studies on these new scaffolds.

#### 2.2.1. Structure Activity Relationship Study

To the best of our knowledge, the existence of *ortho*-position in the aryl moiety for our rationalized compounds has been correlated crucially to this higher selectivity. Results depicted that the structure–activity relationship of our synthesized targeted compounds are as following (Figure 2):

Occupying the *ortho*-position with lipophilic, as well as more of the electron-donating group, i.e., **5g** (Ar = 2-MeO-phenyl, Ki values 0.3 and 3.7 μM respectively, against hCA IX and XII) has showed excellent selectivity.

The replacement of this group with a bromine atom, such as **5b** (Ar = 2-Br-phenyl, Ki values = 0.4 and 4.6 μM, respectively) has also shown an experienced selective inhibition. This might be attributed to the advantages of electron donating (+M) rather than withdrawing (-I) for such an atom.

Similarly, the *ortho*-2,6-dichloro **5d** (Ar = 2,6-diCl-phenyl, Ki values = 0.7 and 4.9 μM, respectively) has also presented desirable selectivity with the consideration of higher (+M) for the bromine than the chlorine atom, albeit with the existence of two chlorine atoms. 

Changing the methoxy group into a hydrophilic one, i.e., **5e** (Ar =2-HO-phenyl, Ki values = 0.8 and 5.2 μM, respectively) has likely displayed acceptable selectivity.

Installment of strong electron withdrawing atom at the same position or alteration of the aryl group to hetero-aryl one, i.e., **5j** (Ar =2-NO_2_-phenyl, Ki value = 1.1 and 6.2 μM, respectively) and **5n** (Ar = thienyl, Ki value = 1.3 and 6.4 μM, respectively) has offered a reasonable selectivity.

Attempting of exploring the *para* position for such hybrids seemed unlikely to be useful for this purpose, such as compound **5f** (Ar = 4-HO-phenyl) which had a 10-fold lower inhibitory activity on hCA IX (Ki = 8.4 μM) in comparison with **5e** (Ar = 2-HO-phenyl, Ki = 0.8 μM). Similarly, **5h** (Ar = 4-MeO-phenyl, Ki = 7.4 μM) displayed 22-fold less reactivity in comparison to the relevant *ortho*-derivative **5g** (Ar = 2-MeO-phenyl, Ki = 0.3 μM).

#### 2.2.2. In Vitro Anticancer Activity

The antiproliferative activity of **5b**, **5d**, **5e**, **5g**, **5j** and **5n** against four human cancer cell lines including pancreatic cancer cell line (Panc-1), breast cancer cell line (MCF-7), colon cancer cell line (HT-29) and epithelial cancer cell line (A-549) using MTT assay and doxorubicin was applied as the reference compound [22,23]. Graph Pad Prism software (Graph Pad Software, San Diego, CA, USA) was used to calculate the median inhibition concentration (IC_50_) and results are recorded in Table 2.

The *o*-methoxy derivative **5g** (Ar = 2-Meo-phenyl) was the most potent of all tested derivatives, with mean GI_50_ value of 2.3 µM against the four cell lines, in comparison with the reference doxorubicin (GI_50_ = 1.1 µM). Compound **5b** (Ar = 2-Br-phenyl) showed higher antiproliferative activity compared to **5d** (Ar = 2,6-dichlorophenyl) with GI_50_ of 2.8 and 3.5 µM, respectively, against the tested cell lines, suggesting the importance of the *ortho*-methoxy group for the antiproliferative activity, Table 2.

Despite having a 10-fold lower efficacy than acetazolamide in enzymatic assays and a 2-fold lower efficacy than doxorubicin in cellular assays, the newly synthesised compounds **5b**, **5d**, **5e**, **5g**, **5j** and **5n** could serve as a starting point for future development of more potent derivatives.

The *o*-methoxy derivative **5g** (Ar = 2-Meo-phenyl) was the most potent of all tested derivatives, with mean GI_50_ value of 2.3 µM against the four cell lines, in comparison to the reference doxorubicin (GI_50_ = 1.1 µM). Compound **5b** (Ar = 2-Br-phenyl) showed higher antiproliferative activity compared to **5d** (Ar = 2,6-dichlorophenyl) with GI_50_ of 2.8 and 3.5 µM, respectively, against the tested cell lines, suggesting the importance of the *ortho*-methoxy group for the antiproliferative activity, Table 2.

### 2.3. Docking Study

Carbonic anhydrases are a ubiquitous metalloenzyme family containing zinc in their active sites. CA-IX is a well-known tumor-associated isoenzyme which is highly overexpressed in different types of cancers, with a limited number in normal tissues [24]. Thus, it is considered a promising drug target for treating cancer. Inspired by the highest inhibitory activity exerted by some of our compounds **5a**–**n** against CA-IX over the other CA isoforms, a docking simulation was performed in order to better understand the molecular mechanisms of inhibition and to determine the binding pattern and the key interactions of the newly synthesized inhibitors within the catalytic active site of CA-IX. It is important to know that the catalytic site of CA IX is comprised of two distinct regions with a Zn^2+^ atom located at the bottom of the active site. The first region is a hydrophobic defined by Leu-91, Val-121, Val-131, Leu-135, Leu-141, Val-143, Leu-198, and Pro-202 amino acid residues while the second region is a hydrophilic identified by Asn-62, His-64, Ser-65, Gla-67, Thr-69, and Gln-92 residues [25].

The docking process was conducted using LibDock tool embedded in the Discovery Studio software 2.5 (San Diego, CA, USA). The 3D crystal structure of CA-IX enzyme (PDB ID: 5FL4, resolution = 1.82 Ǻ) in complex with 2-thiophene-sulfonamide ligand (**9FK**), which acts as a highly efficient inhibitor, has been retrieved from Protein Data Bank (https://www.rcsb.org/structure/5FL4. Accessed on 10 June 2021) [26]. The CA-IX protein structure is a dimer where only one chain was used for the docking study. The amino acid residues within a distance of 10 Å around the co-crystallized ligand in the active pocket were isolated and the key interactions and ligands orientation were analyzed. Ligands and protein files were prepared according to the previous reports [27,28]. The 2D and 3D binding modes of **9FK** and compounds **5a**–**n** were visualized using Discovery Studio Visualizer [29].

**Figure 3:**
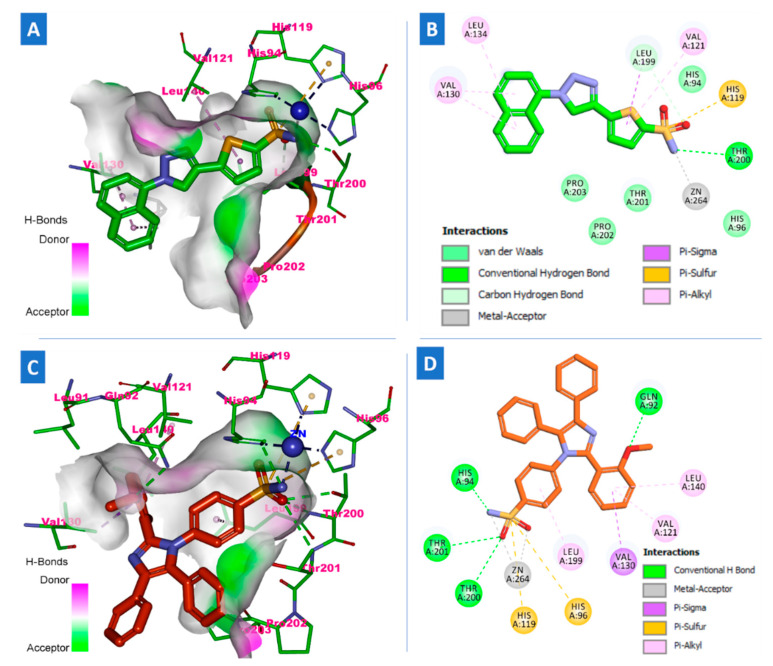
Comparison of docking and 2/3D binding modes of compound **5g** (stick with carbons colored in orange) and co-crystallized inhibitor **9FK** (stick with carbons colored in green) within the catalytic active site of CA-IX isoenzyme (PDB code: 5FL4); (**A**) 3D binding mode of the co-crystallized ligand **9FK** into active site of CA-IX isoenzyme; (**B**) 2D docking mode of **9FK** showing different types of interactions inside the active site of CA-IX isoenzyme; (**C**) 3D binding mode of compound **5g** into active site of CA-IX isoenzyme; (**D**) 2D docking mode of **5g** showing different types of interactions inside the active site of CA-IX isoenzyme. Zn^2+^ cation is presented as a blue sphere in coordination with His94, His96, His119 and -NHSO_2_ shown in dashed dark blue lines. H-bonds are represented as dashed green lines. All hydrogens were removed for the purpose of clarity.

**Figure 4:**
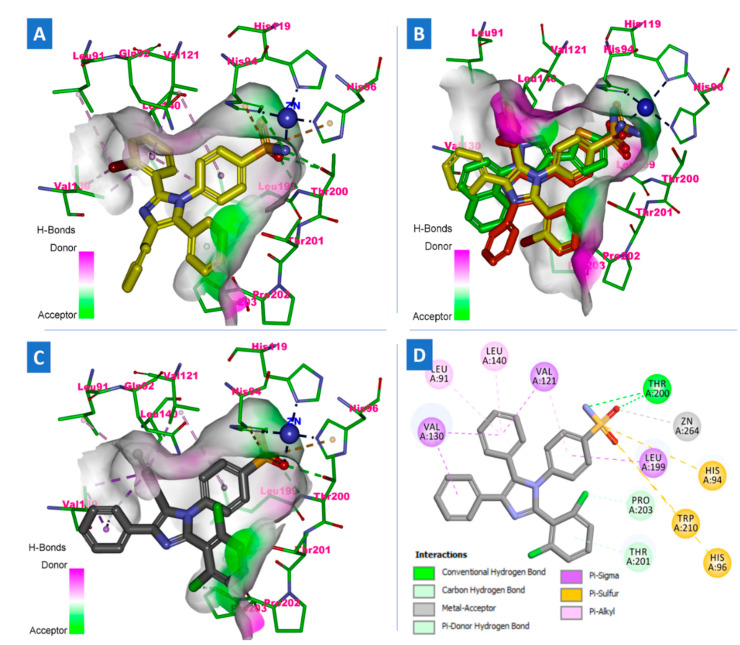
Comparison of docking and 2/3D binding modes of compound **5b** (stick with carbons colored in yellow) and compound **5d** (stick with carbons colored in grey) within the catalytic active site of CA-IX isoenzyme (PDB code: 5FL4); (**A**) 3D binding mode of compound **5b** into active site of CA-IX isoenzyme; (**B**) the superimposition of **5g**, **5b** and **9FK** docked poses within the catalytic active site of CA-IX isoenzyme; (**C**) 3D binding mode of compound **5d** into active site of CA-IX isoenzyme; (**D**) 2D docking mode of **5d** showing different types of interactions inside the active site of CA-IX isoenzyme. Zn^2+^ cation is presented as a blue sphere in coordination with His94, His96, His119 and -NHSO_2_ shown in dashed dark blue lines. H-bonds were represented as dashed green lines. All hydrogens were removed for the purpose of clarity.

**Figure 5:**
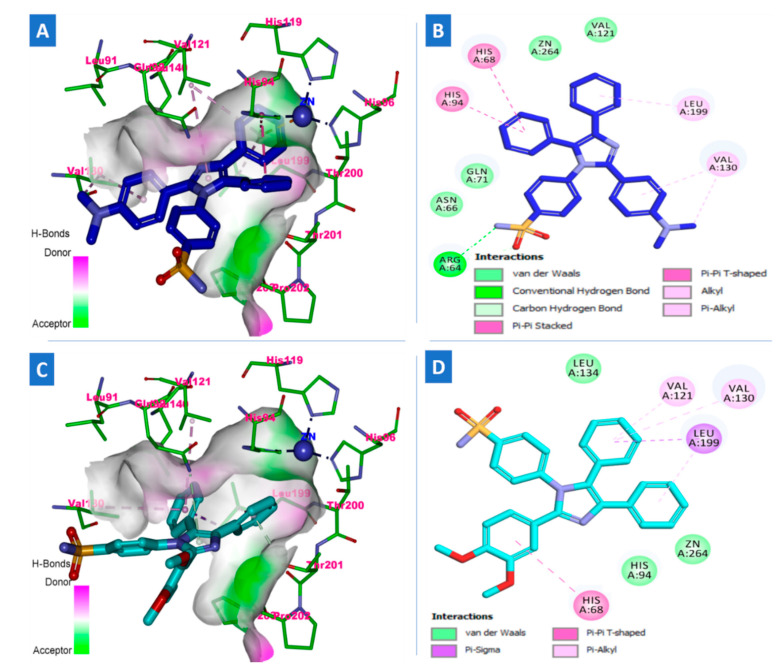
The docking and 2/3D binding modes of compound **5k** (stick with carbons colored in blue) and compound **5i** (stick with carbons colored in cyan) within the catalytic active site of CA-IX isoenzyme (PDB code: 5FL4); (**A**) 3D binding mode of compound **5k** into active site of CA-IX isoenzyme; (**B**) 2D docking mode of **5i** showing different types of interactions inside the active site of CA-IX isoenzyme; (**C**) 3D binding mode of compound **5i** into active site of CA-IX isoenzyme; (**D**) 2D docking mode of **5i** showing different types of interactions inside the active site of CA-IX isoenzyme. Zn^2+^ cation is presented as a blue sphere in coordination with His94, His96, His119 and -NHSO_2_ shown in dashed dark blue lines. H-bonds are represented as dashed green lines. All hydrogens were removed for the purpose of clarity.

**Figure 6:**
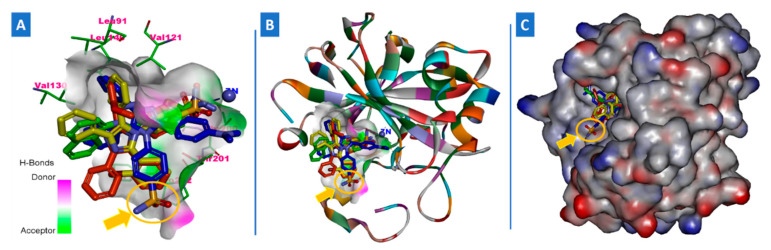
Overlay of the docked poses **5d**, **5k**, **5i** and the co-crystallized ligand **9FK** into the catalytic active site of CA-IX isoenzyme (PDB code: 5FL4). The protein is represented as: (**A**) solid surface colored according to atom charges; (**B**) secondary structure displayed in a solid ribbon (cartoon) style; (**C**) the window shows 3D magnification of superimposition of the four docked poses within the catalytic active site of CA-IX isoenzyme. The poses were rendered as stick model colored in green (**9FK**), blue (**5d**), orange (**5k**) and yellow (**5i**). Zn^2+^ cation is presented as a blue sphere. H-bonds are represented as dashed green lines. All hydrogens were removed for the purpose of clarity.

The docking results of some representative compounds (**5g**, **5b**, **5d**, **5k**, and **5i**) selected according to the activity range (high, moderate, and low) were analyzed and depicted in Figure 3, Figure 4, Figure 5 and Figure 6 in comparison with the co-crystalized sulfonamide ligand **9FK**. Interestingly, the docking results were in agreement with the results of the in vitro CA-IX inhibitory assay. It is well-reported that sulfonamide or sulfamate moiety is a crucial and essential structural feature in potent and specific inhibitors of CA-IX inhibitors where it coordinates the zinc ion in active site and inhibits the enzymatic activity [30,31,32,33,34]. Therefore, the new compounds contain a sulfonamide as a core component owing to their significant inhibitory activity against CA-IX isoform. Inspection of the binding patterns of compounds **5g** and **5b** with the highest activity (IC_50_ = 0.32 and 0.36 µM, respectively) revealed that they bind very similarly within the CA-IX active pocket with a very similar binding mechanism and orientation to that of co-crystalized ligand **9FK**. The deprotonated sulfonamide moiety was positioned toward the hydrophobic region in the active site, where its nitrogen atom formed a coordination bond with the Zn^2+^ cation such as **9FK** and became involved in a H-bond with His94 amino acid. Moreover, the two oxygen atoms of the SO_2_ group in compound **5g** formed a network of critical H-bonding interactions with key residues in the active site, including Leu199 Thr200 and Thr201 in addition to one more optimal H-bond between the methoxy group and Gln92 amino acid, exceeding the number of H-bond interactions formed by ligand **9FK**. However, the other two unsubstituted phenyl groups of **5g**, equivalent to the naphthyl moiety of **9FK**, were positioned at the entrance of the catalytic active site cleft forming some van der Waals and hydrophobic interactions with Pro202 and Pro203 residues. The 2D and 3D binding interactions of **5g** and **9FK** within the active site of CA-IX enzyme were shown in Figure 3A–D.

In Figure 4A, compound **5b** showed almost an identical binding mode engaging similar interactions to that exerted by **5g**. The sulfonamide moiety was positioned towards the bottom of the active site where a coordination bond was formed in addition to the same network H-bonding interactions with the well-known key amino acid residues, such as His94, Leu199, Thr200 and Thr201. The absence of the crucial H-bond with Gln92 emerged as a result of replacing the methoxy group with a bromo moiety in **5b**, owing to the very slight difference in activities between the two superior compounds **5g** and **5b**. The superimposition of **9FK**, **5g** and **5b**, as shown in Figure 4B, suggested a similar orientation and binding pattern of the three ligands, explaining the superior activity with the same inhibition mechanism inside CA-IX catalytic pocket. Meanwhile, the docking of **5d** revealed an exhibition of disposition similar to **5g** forming a coordination bond with Zn^2+^ ion through the one oxygen of sulfonamide instead of the nitrogen atom. Additionally, the absence of some key H-bonds with Gln92, His94, Leu199 and Thr201 residues was observed. In addition, the change in the methoxy group with dichloro atoms, i.e., **5d**, resulted in some clashes in the hydrophobic region of the CA-IX active site. These reasons can obviously clarify the moderate inhibitory activity of **5d** (IC_50_ = 0.72 µM) compared to the remarkable activity of **5g** (IC_50_ = 0.32 µM).

Investigation of docking results of compounds **5k** and **5i,** which had minimal potency (IC_50_ = 9.3 and 9.2 μM), revealed a completely inverse orientation and binding mode compared to the co-crystallized ligand **9FK** or the active compounds such as **5g** and **5b**. It was found that the sulfonamide moiety was pushed outside the active site in the opposite side of the Zn^2+^ cation location and the two unsubstituted phenyl groups were occupied the hydrophobic region near the bottom of the catalytic active site. This inverse disposition resulted in a real loss of the crucial H-bonding interactions with the key-amino acids (His94, Leu199, Thr200 and Thr201) and the important coordination bond with Zn^2+^ cation which in turn caused the remarkable reduction in the inhibitory activity of the corresponding ligands **5k** and **5i**, Figure 5A–D. It was worth noting that these two respected derivatives, **5k** and **5i,** bearing the bulkiest substituents on one of the phenyl groups (dimethylamine or dimethoxy, respectively) in the series. Based on that, it was conceptualized that these bulky groups could not be accommodated within the active site and did not coordinate with the Zn^2+^ ion. Accordingly, these groups preferred to protrude outside the catalytic active site owing to the sharp decrease in their activity in comparison with **9FK**, **5g** and **5b**. Figure 5D showed this disparity clearly through the superimposition of **5k** and the co-crystallized ligand **9FK**.

To conclude our study, and pointing out the variation in the biological results in a collective way, the top docking poses **5g**, **5b** and **9FK** were overlaid into CA-IX catalytic active pocket and displayed in Figure 6A–C. Obviously, the poses **5g**, **5b** and **9FK** were found to fit nicely and demonstrated good shapes complementarity with the active site of the enzyme, while compounds **5k** exhibited a completely converse orientation without forming the essential interactions required for the CA-IX inhibition mechanism. Taken together, the docking study provided a clear explanation for the in vitro CA-IX assay results and pointed out the right positioning, crucial structural features and key-interactions of potent CA-IX inhibitors that greatly contribute to designing and developing potential leads for CA-IX as a promising anticancer drug target.

## 3. Materials and Methods

### 3.1. Chemistry

General Details: See Appendix A.

#### General Procedure for Synthesis of 4-(4,5-Diphenyl-2-(substituted benzene)-1H-imidazol-1-yl)benzene Sulfonamides **5a–n**

In a rounded 50 mL flask, benzil **1** (2.10 g, 5mmol) was added to an equivalent amount of sulfanilamide **2** (1.72 g, 10 mmol), ammonium acetate **3** (0.77 g, 10 mmol), the appropriate aldehyde **4a–n** (10 mmol), and diethyl ammonium hydrogen sulfate (0.51 g, 3 mmol). The mixture was refluxed in an oil bath with stirring at 110 °C for about 15 min. The reaction was monitored with TLC using chloroform: ethanol (9:1). The reaction mixture was poured into iced water and the resulting precipitate was filtered off, washed with water, and then dried. The crude product was crystallized from ethanol.

*4-(2,4,5-Triphenyl-1H-imidazol-1-yl)benzene sulfonamide* **5a**

Yellowish white solid, Yield: 70%; m.p.: 210–212 °C; ^1^H NMR (400 MHz, DMSO) *δ*: 7.20–7.30 (m, 5H, ArH), 7.34–7.46 (m, 12H, ArH), 7.54 (d, *J* = 7.1 Hz, 2H, ArH), 7.76 (d, *J* = 7.1 Hz, 2H, NH_2_) ppm; ^13^C NMR (100 MHz, DMSO) *δ*: 146.77, 144.60, 139.81, 137.71, 134.68, 131.67, 131.55, 130.67, 130.59, 129.80, 129.10, 129.02, 128.74, 128.64, 127.06, 126.93, 126.89 ppm. Anal. Calcd for C_27_H_21_N_3_O_2_S (451.14): C, 71.82; H, 4.69; N, 9.31. Found: C, 71.86; H, 4.67; N, 9.33%.

*4-(2-(2-Bromophenyl)-4,5-diphenyl-1H-imidazol-1-yl)benzene sulfonamide* **5b**

White solid, Yield: 78%; m.p.: 220–222 °C; ^1^H NMR (400 MHz, DMSO) *δ*: 7.20–7.30 (m, 3H, ArH), 7.34–7.38 (m, 3H, ArH), 7.43–7.54 (m, 4H, ArH), 7.61–7.67 (m, 6H, ArH), 7.68–7.96 (4H, 2ArH + NH_2_) ppm; ^13^C NMR (100 MHz, DMSO) *δ*: 146.13, 144.20, 138.82, 137.36, 136.38, 135.93, 134.45, 133.69, 132.88, 132.79, 131.92, 131.45, 130.03, 129.93, 129.26, 129.18, 128.86, 128.67, 127.94, 127.88, 127.13, 126.92, 126.53, 124.32, 121.72, 113.03 ppm. Anal. Calcd for C_27_H_20_BrN_3_O_2_S (529.05): C, 61.14; H, 3.80; N, 7.92. Found: C, 61.11; H, 3.78; N, 7.89%.

*4-(2-(4-Chlorophenyl)-4,5-diphenyl-1H-imidazol-1-yl)benzene sulfonamide* **5c**

Yellowish solid, Yield: 75%; m.p.: 230–232 °C; ^1^H NMR (400 MHz, DMSO) *δ*: 7.20–7.29 (m, 5H, ArH), 7.35–7.40 (m, 7H, ArH), 7.45–7.53 (m, 6H, ArH), 7.77 (d, *J* = 8.1 Hz, 2H, NH_2_) ppm; ^13^C NMR (100 MHz, DMSO) *δ*: 145.64, 144.73, 139.56, 137.88, 134.51, 133.92, 131.81, 131.64, 130.64, 130.42, 129.76, 129.48, 129.23, 129.11, 128.86, 128.65, 127.14, 127.05, 126.88 ppm. Anal. Calcd for C_27_H_20_ClN_3_O_2_S (485.10): C, 66.73; H, 4.15; N, 8.65. Found: C, 66.70; H, 4.12; N, 8.62%.

*4-(2-(2,6-Dichlorophenyl)-4,5-diphenyl-1H-imidazol-1-yl)benzene sulfonamide* **5d**

Yellow solid, Yield: 80%; m.p.: 214–216 °C; ^1^H NMR (400 MHz, DMSO) *δ*: 7.45–7.49 (m, 7H, ArH), 7.61–7.65 (m, 4H, 2ArH+ NH_2_), 7.78–7.80 (m, 8H, 8ArH) ppm.^13^C NMR (100 MHz, DMSO) *δ*: 195.24, 135.86, 132.83, 131.98, 131.17, 129.97, 129.88, 129.00, 128.52, 127.93, 127.43, 126.91, 113.08, 111.91, 111.54 ppm. Anal. Calcd for C_27_H_19_Cl_2_N_3_O_2_S (519.06): C, 62.31; H, 3.68; N, 8.07. Found: C, 62.33; H, 3.70; N, 8.10%.

*4-(2-(2-Hydroxyphenyl)-4,5-diphenyl-1H-imidazol-1-yl)benzene sulfonamide* **5e**

Whitish solid, Yield: 74%; m.p.: 250–252 °C; ^1^H NMR (400 MHz, DMSO) δ: 5.90 (s, 2H, NH_2_), 6.72–6.76 (m, 4H, ArH), 7.00–7.02 (m, 2H, ArH), 7.40–7.64 (m, 12H, ArH), 8.42 (broad s, 1H, OH) ppm; ^13^C NMR (100 MHz, DMSO) *δ*: 165.59, 155.50, 139.09, 135.07, 134.64, 131.55, 129.39, 128.76, 128.44, 128.01, 127.31, 126.68, 122.85, 119.25, 115.76 ppm. Anal. Calcd for C_27_H_21_N_3_O_2_S (467.13): C, 69.36; H, 4.53; N, 8.99. Found: C, 69.39; H, 4.57; N, 9.02%.

*4-(2-(4-Hydroxyphenyl)-4,5-diphenyl-1H-imidazol-1-yl)benzene sulfonamide* **5f**

Pale yellow solid, Yield: 68%; m.p.: 240–242 °C; ^1^H NMR (400 MHz, DMSO) *δ*: 6.73 (d, *J* = 8.6 Hz, 2H, ArH), 7.18–7.27 (m, 7H, ArH), 7.40 (d, *J* = 8.4, 5H, ArH), 7.48–7.54 (m, 4H, ArH), 7.78 (d *J* = 8.4, 2H, NH_2_), 9.76 (s, 1H, OH) ppm; ^13^C NMR (100 MHz, DMSO) *δ*: 158.29, 147.25, 144.42, 140.08, 137.39, 134.84, 131.67, 130.84, 130.81, 130.56, 129.78, 129.04, 128.57, 126.92, 121.49, 115.62 ppm. Anal. Calcd for C_27_H_21_N_3_O_2_S (467.13): C, 69.36; H, 4.53; N, 8.99. Found: C, 69.38; H, 4.56; N, 9.01%.

*4-(2-(2-Methoxyphenyl)-4,5-diphenyl-1H-imidazol-1-yl)benzene sulfonamide* **5g**

White solid, Yield: 82%; m.p.: 268–270 °C; ^1^H NMR (400 MHz, DMSO) *δ*: 6.68–7.06 (m, 2H, ArH), 7.17–7.27 (m, 11H, ArH), 7.34–7.41 (m, 4H, ArH), 7.48–7.57 (m, 3H, ArH), 7.61–7.64 (m, 2H, NH_2_); ^13^C NMR (100 MHz, DMSO) *δ*: 156.95, 145.40, 143.64, 139.78, 137.54, 134.80, 132.53, 131.62, 131.48, 130.70, 129.95, 129.19, 129.01, 128.61, 128.47, 126.90, 126.13, 120.89, 120.07, 111.67, 55.09 ppm. Anal. Calcd for C_28_H_23_N_3_O_3_S (481.15): C, 69.84; H, 4.81; N, 8.73. Found: C, 69.87; H, 4.82; N, 8.72%.

*4-(2-(4-Methoxyphenyl)-4,5-diphenyl-1H-imidazol-1-yl)benzene sulfonamide* **5h**

Yellow solid, Yield: 85%; m.p.: 235–237 °C; ^1^H NMR (400 MHz, DMSO) *δ*: 3.76 (s, 3H, OCH_3_), 6.88 (d, *J* = 8.2 Hz, 2H, ArH), 7.18–7.33 (m, 12H, ArH), 7.47 (d, *J* = 20.8 Hz and 7.9 Hz, 4H, ArH), 7.74 (d, *J* = 8.1 Hz, 2H, NH_2_) ppm; ^13^C NMR (100 MHz, DMSO) *δ*: 159.89, 146.72, 144.47, 139.94, 137.40, 134.76, 131.66, 131.11, 130.70, 130.40, 129.84, 129.08, 128.61, 126.92, 126.83, 123.04, 114.24, 55.66 ppm. Anal. Calcd for C_28_H_23_N_3_O_3_S (481.15): C, 69.84; H, 4.81; N, 8.73. Found: C, 69.87; H, 4.87; N, 8.77%.

*4-(2-(3,4-Dimethoxyphenyl)-4,5-diphenyl-1H-imidazol-1-yl)benzene sulfonamide* **5i**

Pale yellow solid, Yield: 73%; m.p.: 260–262 °C; ^1^H NMR (400 MHz, DMSO) *δ*: 3.52 (s, 3H, CH_3_), 3.76 (s, 3H, CH_3_), 6.84–7.05 (3H, ArH), 7.26–7.35 (9H, ArH), 7.46–7.53 (m, 5H, ArH), 7.77–7.79 (d, *J* = 8.5 Hz, 2H, NH_2_) ppm; ^13^C NMR (100 MHz, DMSO) *δ*: 149.57, 148.53, 146.68, 144.59, 140.10, 137.41, 134.75, 131.66, 131.14, 130.66, 129.97, 129.10, 128.62, 126.94, 126.90, 122.98, 121.81, 112.53, 112.00, 55.97, 55.66 ppm. Anal. Calcd for C_29_H_25_N_3_O_4_S (511.16): C, 68.08; H, 4.93; N, 8.21. Found: C, 68.12; H, 4.97; N, 8.24%.

*4-(2-(2-Nitrophenyl)-4,5-diphenyl-1H-imidazol-1-yl)benzene sulfonamide* **5j**

Yellowish solid, Yield: 86%; m.p.: 280–282 °C; ^1^H NMR (400 MHz, DMSO) *δ*: 7.18–7.38 (m, 10H, ArH), 7.45–7.47 (m, 2H, ArH), 7.64–7.76 (m, 5H, ArH), 7.90–8.16 (m, 3H, 1ArH + NH_2_) ppm. Anal. Calcd for C_27_H_20_N_4_O_4_S (496.12): C, 65.31; H, 4.06; N, 11.28. Found: C, 65.35; H, 4.10; N, 11.24%.

*4-(2-(4-(Dimethylamino)phenyl)-4,5-diphenyl-1H-imidazol-1-yl)benzene sulfonamide* **5k**

White solid, Yield: 76%; m.p.: 224–226 °C; ^1^H NMR (400 MHz, DMSO) *δ*: 2.88 (s, 3H, CH_3_), 3.03 (s, 3H, CH_3_), 6.58–6.79 (m, 3H, ArH), 7.19–7.49 (m, 15H, ArH), 7.68–7.75 (m, 2H, NH_2_) ppm.^13^C NMR (100 MHz, DMSO) *δ*: 152.32, 150.63, 147.59, 144.25, 140.31, 137.29, 134.83, 132.05, 131.66, 130.81, 130.71, 130.61, 129.96, 129.83, 129.02, 128.56, 127.87, 126.89, 117.73, 113.09, 111.87, 111.56, 39.24 ppm. Anal. Calcd for C_29_H_26_N_4_O_2_S (494.18): C, 70.42; H, 5.30; N, 11.33. Found: C, 70.46; H, 5.33; N, 11.34%.

*4-(4,5-Diphenyl-1-(4-sulfamoylphenyl)-1H-imidazol-2-yl)benzoic acid* **5l**

Pale yellow solid, Yield: 90%; m.p.: 200–202 °C; ^1^H NMR (400 MHz, DMSO) *δ*: 7.20–7.37 (m, 5H, ArH), 7.40–7.51 (m, 4H, ArH), 7.57–7.69 (m, 4H, ArH), 7.77–6.88 (m, 3H, ArH), 7.93–8.23 (m, 5H, 3ArH+ NH_2_ + OH) ppm; ^13^C NMR (100 MHz, DMSO) δ 167.25, 145.71, 144.76, 139.56, 138.16, 135.96, 134.44, 132.21, 131.64, 130.94, 130.29, 130.03, 129.96, 129.77, 129.67, 129.31, 129.14, 128.88, 128.69, 127.23, 127.10, 126.90 ppm. Anal. Calcd for C_28_H_21_N_3_O_4_S (495.13): C, 67.87; H, 4.27; N, 8.48. Found: C, 67.90; H, 4.30; N, 8.50%.

*Ethyl 4-(4,5-diphenyl-1-(4-sulfamoylphenyl)-1H-imidazol-2-yl)benzoate* **5m**

White solid, Yield: 88%; m.p.: 216–218 °C; ^1^H NMR (DMSO, 400 MHz) *δ*: 1.07 (t, *J* = 7.0 Hz, 3H, CH_3_), 3.36–3.48 (m, 2H, CH_2_), 7.21–7.88 (m, 20H, 18 ArH+ NH_2_) ppm.^13^C NMR (100 MHz, DMSO) *δ*: 167.26, 145.75, 144.79, 139.58, 138.18, 134.44, 132.22, 131.65, 131.08, 130.32, 129.76, 129.63, 129.28, 129.12, 128.86, 128.66, 127.21, 127.07, 126.91, 56.53, 18.95 ppm. Anal. Calcd for C_30_H_25_N_3_O_4_S (523.16): C, 68.82; H, 4.81; N, 8.03. Found: C, 68.86; H, 4.83; N, 7.99%.

*4-(4,5-Diphenyl-2-(thiophen-2-yl)-1H-imidazol-1-yl)benzene sulfonamide* **5n**

Yellowish white solid, Yield: 92%; m.p.: 290–292 °C; ^1^H NMR (400 MHz, DMSO) *δ*: 6.58 (d, *J* = 3.2, 1H, thienyl-H), 6.95–7.32 (m, 10H, 3H thienyl and 7ArH) 7.52 (dd, *J* = 17.0 Hz and 7.52 Hz, 4H, ArH), 7.64 (d, *J* = 8.3, 2H, ArH), 7.86 (d, *J* = 8.3, 2H, NH_2_) ppm; ^13^C NMR (100 MHz, DMSO) *δ*: 145.45, 141.80, 139.31, 137.72, 135.93, 134.34, 132.95, 131.59, 130.30, 130.20, 129.99, 129.95, 129.64, 129.22, 129.08, 128.65, 127.92, 127.23, 126.85, 126.39 ppm. Anal. Calcd for C_25_H_19_N_3_O_2_S_2_ (457.09): C, 65.62; H, 4.19; N, 9.18. Found: C, 65.66; H, 4.23; N, 9.22%.

### 3.2. Biology

#### 3.2.1. Carbonic Anhydrase Assay

The ability of imidazoles **5a**–**n** toward inhibition of hCA I, II (cytosolic) as well as hCA IX and XII (transmembrane, tumor-associated isoforms) using stopped-flow CO_2_ hydrase assay was evaluated [19]. See Appendix A.

#### 3.2.2. Cell Viability Assay

Cell viability assay was carried out using human mammary gland epithelial cell line (MCF-10A) [20]. MCF-10A cells were incubated with compounds **5b**, **5d**, **5e**, **5g**, **5j** and **5n** for 4 days and MTT assay [21] was used to determine the viability of cells. See Appendix A.

#### 3.2.3. Antiproliferative Activity

The antiproliferative activity of **5b**, **5d**, **5e**, **5g**, **5j** and **5n** against four human cancer cell lines including pancreatic cancer cell line (Panc-1), breast cancer cell line (MCF-7), colon cancer cell line (HT-29) and epithelial cancer cell line (A-549) using MTT assay and doxorubicin was applied as the reference compound [22,23]. See Appendix A.

## 4. Conclusions

A novel tranche of triaryl imidazole derivatives bearing benzene sulfonamide moiety has been rationalized and synthesized. The ability of imidazoles **5a**–**n** toward inhibition of hCA I, II (cytosolic) as well as hCA IX and XII (transmembrane, tumor-associated isoforms) using stopped-flow CO2 hydrase assay was evaluated. Results displayed that our targets **5a**–**n** had selective inhibition of hCA IX and XII in comparable with hCA I and II in the micromolar range. The most potent and selective hCA IX inhibitors **5g**, **5b**, **5d**, **5e**, **5j** and **5n** with Ki values of 0.3, 0.4, 0.7, 0.8, 1.1 and 1.3 µM, respectively, were further assessed for their anti-proliferative activity against four different cancer cell lines using MTT assay in comparison to doxorubicin as reference standard. Anti-proliferative assay displayed that compound **5g** showed higher inhibitory activity other tested ones in the order of **5g** > **5b** > **5d** > **5e** > **5j** > **5n**. Molecular docking study demonstrated high docking scores and good binding interactions of the most active compounds **5g** and **5b** within the hCA-IX active pocket with adoption of similar orientation to that of co-crystalized ligand **9FK**. This concludes our assumption of the impact of electronic rich environment of ortho-position rather than other ones (meta- and para-) and its impact as a pivotal feature for developing highly selective hCA IX and XII inhibitors. Overall, our novel hybrids have opened the door to a new significant approach for the imidazole scaffold being engaged with benzene sulfonamide via a strategy that appeared to be little studied up until now. Indeed, compounds **5g** and **5b** are likely to be prospective lead candidates for further consideration and optimization to explore more about the structure–activity relationship and release novel, potent hCA IX and XII selective inhibitors agent.

## Figures and Tables

**Table 1:** Inhibition of hCA isoforms **I**, **II**, **IX** and **XII** with compounds **5a**–**n** and acetazolamide (AAZ).

Compound	hCA I	hCA II	hCA IX	hCA XII	Selectivity Ratio
					hCA XII/ hCA IX
**5a**	>100	>100	2.9	5.5	1.90
**5b**	>100	>100	0.4	4.6	11.50
**5c**	>100	>100	8.2	3.7	0.45
**5d**	>100	>100	0.7	4.9	7.00
**5e**	>100	>100	0.8	5.2	6.50
**5f**	>100	>100	8.4	7.1	0.90
**5g**	>100	>100	0.3	3.7	12.00
**5h**	>100	>100	7.4	3.9	0.50
**5i**	>100	>100	9.2	6.6	0.70
**5j**	>100	>100	1.1	6.2	5.60
**5k**	>100	>100	9.3	6.8	0.70
**5l**	>100	>100	8.5	6.8	0.80
**5m**	>100	>100	8.8	6.2	0.70
**5n**	>100	>100	1.3	6.4	5.00
AAZ	0.25	0.012	0.026	0.006	0.20

**Table 2:** Antiproliferative activity of compounds **5b**, **5d**, **5e**, **5g**, **5j**, **5n** and Doxorubicin.

Comp.	Cell Viability %	Antiproliferative Activity IC_50_ ± SEM (µM)				
		A-549	MCF-7	Panc-1	HT-29	Average
**5b**	91	2.8 ± 0.5	2.7 ± 0.4	2.5 ± 0.2	3.1 ± 0.1	2.8
**5d**	87	3.1 ± 0.6	3.9 ± 0.4	3.8 ± 0.3	3.1 ± 0.4	3.5
**5e**	86	4.4 ± 0.7	3.6 ± 0.8	3.7 ± 0.5	3.9 ± 0.6	3.9
**5g**	90	2.4 ± 0.5	2.1 ± 0.2	2.2 ± 0.4	2.4 ± 0.4	2.3
**5j**	88	4.6 ± 0.6	3.6 ± 0.2	4.6 ± 0.9	4.8 ± 0.4	4.4
**5n**	90	4.7 ± 0.6	4.6 ± 0.6	4.8 ± 0.3	4.8 ± 0.2	4.7
Doxorubicin	-	1.2 ± 0.8	0.9± 0.6	1.4 ±0.6	1.0 ± 0.8	1.1

## Data Availability

Not applicable.

## Sample Availability

Samples of the compounds are not available from the authors.

## Appendix A. Experimental

### Appendix A.1. Chemistry

General Details: Melting points were determined with a Gallenkamp (London, UK) melting point apparatus and are uncorrected. Infrared spectra (νmax) were recorded on Bruker Vector, 22FT-IR [Fourier Transform Infrared (FTIR), Germany] spectrometer. ^1^H NMR and ^13^C NMR spectra were recorded at room temperature on a Varian Gemini-400 (400 MHz, Foster City, CA, USA) spectrometer operating at 400 MHz for proton and 100 MHz for carbon nuclei using dimethylsulphoxide and/or (DMSO/D_2_O) as solvent and tetramethylsilane (*TMS*) as an internal standard (chemical shift in δ, ppm). The signal due to residual [(CH_3_)_2_SO] appearing at δH 2.50 and the central resonance of the [(CD_3_)_2_SO] “multiplet” appearing at δ C 39.0 were used to reference ^1^H and ^13^C NMR spectra, respectively. ^1^H NMR data are recorded as follows: chemical shift (δ) [multiplicity, coupling constant(s) J (Hz), relative integral] where multiplicity is defined as s = singlet; d = doublet; t = triplet; q = quartet; and m = multiplet or combinations of the above. High-resolution measurements were conducted on a time-of-flight instrument. High-resolution EI mass spectra were recorded on a magnetic-sector machine. All the results of elemental analyses corresponded to the calculated values within experimental error. Progress of the reaction was monitored by thin-layer chromatography (TLC) using precoated TLC sheets with Ultraviolet (UV) fluorescent silica gel (Merck 60F254) and spots were visualized by iodine vapours or irradiation with UV light (254 nm). All the starting materials and reagents were generally commercially available and purchased from Sigma-Aldrich (Kenilworth, NJ, USA) or Lancaster Synthesis Corporation (Lancashire, UK).

### Appendix A.2. In Vitro Carbonic Anhydrase Inhibitory Assay

The carbonic anhydrase catalyzed CO_2_ hydration actions for all new compounds herein reported have were assayed utilizing an instrument of Applied Photophysics stopped-flow. The enzymes are recombinant proteins prepared in our lab. Phenol red (at a concentration of 0.2 mM) has been used as indicator, working at the absorbance maximum of 557 nm, with 20 mM Hepes (pH 7.5) as buffer, and 20 mM Na_2_SO_4_ (for maintaining constant the ionic strength), following the initial rates of the CA-catalyzed CO_2_ hydration reaction for a period of 10–100 s. The CO_2_ concentrations ranged from 1.7 to 17 mM for the determination of the kinetic parameters and inhibition constants. For each inhibitor at least six traces of the initial 5–10% of the reaction have been used for determining the initial velocity. The uncatalyzed rates were determined in the same manner and subtracted from the total observed rates. Stock solutions of inhibitor (0.1 mM) were prepared in distilled-deionized water and dilutions up to 0.01 nM were done thereafter with the assay buffer. Inhibitor and enzyme solutions were preincubated together for 15 min at room temperature prior to assay, in order to allow for the formation of the E-I complex. The inhibition constants were obtained by non-linear least-squares methods using PRISM 3 and the Cheng-Prusoff equation, and represent the mean from at least three different determinations.

### Appendix A.3. Cytotoxic Activity Using MTT Assay and Evaluation of IC_50_

#### Appendix A.3.1. MTT Assay

MTT assay was carried out to study the effect of compounds on mammary epithelial cells (MCF-10A) [20,21]. The medium in which cells were propagated contained Dulbecco’s modified Eagle’s medium (DMEM)/Ham’s F-12 medium (1:1) supplemented with epidermal growth factor (20 ng/mL), hydrocortisone (500 ng/mL), insulin (10 μg/mL), 2 mM glutamine and 10% fetal calf serum. After every 2–3 days, the cells were passaged using trypsin ethylenediamine tetra acetic acid (EDTA). The cells were seeded at a density of 10^4^ cells mL^−1^ in flat-bottomed culture plates containing 96 wells each. After 24 h, medium was removed from the plates and the compounds in (in 0.1% DMSO) were added (in 200 μL medium to yield a final concentration of 0.1% *v*/*v*) to the wells of plates. A single compound was designated with four wells followed by incubation of plates for 96 h at 37 °C. After incubation, medium was removed completely from the plates followed by addition of MTT (0.4 mg/mL in medium) to each well and subsequent incubation of plates for 3 h. MTT (along with the medium) was removed and DMSO (150 μL) was added to each well of the culture plates, followed by vortexing and subsequent measurement of absorbance (at 540 nm) using microplate reader. The data are shown as percentage inhibition of proliferation in comparison with controls containing 0.1% DMSO.

#### Appendix A.3.2. Assay for Antiproliferative Effect

To explore the antiproliferative potential of compounds MTT assay was performed according to previously reported procedure [22,23] using different cell lines To explore the antiproliferative potential of compounds propidium iodide fluorescence assay was performed using different cell lines. To calculate the total nuclear DNA, a fluorescent dye (propidium iodide, PI) is used which can attach to the DNA, thus offering a quick and precise technique. PI cannot pass through the cell membrane and its signal intensity can be considered as directly proportional to quantity of cellular DNA. Cells whose cell membranes are damaged or have changed permeability are counted as dead ones. The assay was performed by seeding the cells of different cell lines at a density of 3000–7500 cells/well (in 200 µL medium) in culture plates followed by incubation for 24 h at 37 °C in humidified 5% CO_2_/95% air atmospheric conditions. The medium was removed; the compounds were added to the plates at 10 µM concentrations (in 0.1% DMSO) in triplicates, followed by incubation for 48 h. DMSO (0.1%) was used as control. After incubation, medium was removed followed by the addition of PI (25 µL, 50µg/mL in water/medium) to each well of the plates. At −80 °C, the plates were allowed to freeze for 24 h, followed by thawing at 25 °C. A fluorometer (Polar-Star BMG Tech, Offenburg, Germany) was used to record the readings at excitation and emission wavelengths of 530 and 620 nm for each well. The percentage cytotoxicity of compounds was calculated using the following formula:

%Cytotoxicity=(Ac−Atc)/Ac×100

where A*tc* = Absorbance of treated cells and A*c* = Absorbance of control. Erlotinib was used as positive control in the assay.

### Appendix A.4. Statistical Analysis

Computerized Prism 5 program was used to statistically analyzed data using one-way ANOVA test followed by Tukey’s as post ANOVA for multiple comparison at *p* ≤ 0.05. Data were presented as mean ± SEM.
